# Down-regulation of 14q32-encoded miRNAs and tumor suppressor role for *miR-654-3p* in papillary thyroid cancer

**DOI:** 10.18632/oncotarget.14162

**Published:** 2016-12-24

**Authors:** Murilo Vieira Geraldo, Helder Imoto Nakaya, Edna Teruko Kimura

**Affiliations:** ^1^ Department of Cell and Developmental Biology, Institute of Biomedical Sciences, University of Sao Paulo, Sao Paulo, Brazil; ^2^ Department of Clinical Analyses and Toxicology, School of Pharmaceutical Sciences, University of Sao Paulo, Sao Paulo, Brazil; ^3^ Department of Structural and Functional Biology, Institute of Biology, State University of Campinas, Campinas, Brazil

**Keywords:** microRNA, papillary thyroid carcinoma, 14q32 region, microTranscriptome, metastasis

## Abstract

Papillary thyroid carcinoma (PTC) is the most prevalent malignant neoplasia of the thyroid gland. A fraction of PTC cases show loss of differentiation and aggressive behavior, with radioiodine therapy resistance and metastasis. Although microRNAs (miRNAs) emerged as promising molecular markers for PTC, their role in the loss of differentiation observed during PTC progression remains to be fully understood. We performed the large-scale analysis of miRNA expression during PTC progression in *BRAFT1799A*-transgenic animals (Tg-Braf) and thyroid cancer cell lines and identified the marked downregulation of several miRNAs from the region 14q32. Data from The Cancer Genome Atlas (TCGA) confirmed the global downregulation of miRNAs from the 14q32 region in human PTC. The regulatory network potentially suppressed by these miRNAs suggests that key cancer-related biological processes such as cell proliferation, adhesion, migration and angiogenesis. Among the downregulated miRNAs, we observed that *miR-654-3p* levels decrease with long-term PTC progression in Tg-Braf mice and inversely correlate with EMT. The *in vitro* restoration of *miR-654-3p* decreased cell proliferation and migration and induced reprogramming of metastasis-related genes, suggesting a tumor suppressor role for this miRNA. In conclusion, we show global downregulation of 14q32-encoded miRNAs in an *in vivo* model of PTC progression. The potential circuitry in which these miRNAs are involved suggests that these miRNAs could play a key role in the pathophysiology of PTC and therefore be relevant for the development of new therapeutic strategies.

## INTRODUCTION

In the last decade, an increasing number of thyroid cancer cases has been observed worldwide. More than 64,300 new cases of thyroid cancer are estimated to be diagnosed in 2016, leading to 1,980 deaths [1] in USA. The most prevalent subtype is papillary thyroid carcinoma (PTC), accounting for 80% of all cases. Although PTCs typically confer good prognoses, about 5% of PTC cases present as aggressive tumors, with radioiodine therapy resistance, lymph node metastatic dissemination and poor prognosis [2]. The most prevalent genetic alterations in thyroid tumors are *BRAFT1799A* mutation and *RET/PTC* chromosomal rearrangements, which have been extensively explored as prognostic markers and therapeutic targets [3–5].

miRNAs are small RNA molecules involved in post-transcriptional regulation of gene expression, mainly through imperfect base pairing with the 3′ untranslated region (UTR) of target messenger RNA (mRNA)[6]. Several studies have implicated differential expression of miRNAs as a promising molecular marker for aggressive and recurrent thyroid cancer [7–11]. Due to a short length and imperfect base-pairing, a single miRNA is predicted to regulate hundreds of target mRNAs. Conversely, several miRNAs can cooperate to regulate a single target. Thus, the contribution of multiple deregulated miRNAs to post-transcriptional regulation in thyroid cancer, particularly regarding aggressive PTC, remains poorly understood.

In this study, we used Tg-Braf mice to assess the micro-transcriptome during PTC progression and identified downregulation of several miRNAs from the 14q32 genomic region. This region spans ∼850 kb and harbors distinct imprinted genes (*DLK1, RTL1, MEG3, MEG8* and *DIO3*), C/D small nucleolar RNAs (*SNORDs*) and more than 50 miRNA genes, many described as tumor suppressors in different types of cancer [12, 13]. The observation that a subset of Temple Syndrome (TS) patients, in which the 14q32 region is partially or completely deleted, present with increased risk of developing differentiated thyroid cancer suggests that some of the genes located in this region may modulate biological processes crucial for thyroid follicular cell transformation [14, 15]. Our *in silico* analysis highlights miRNAs from the 14q32 locus as candidate targets in PTC. Furthermore, we show tumor suppressor properties for *miR-654-3p in vitro*, which could open perspectives for new therapeutic strategies for PTC.

## RESULTS

### Global downregulation of miRNAs from the 14q32 genomic locus in a transgenic mouse model of PTC and human PTC samples

**Figure 1:**
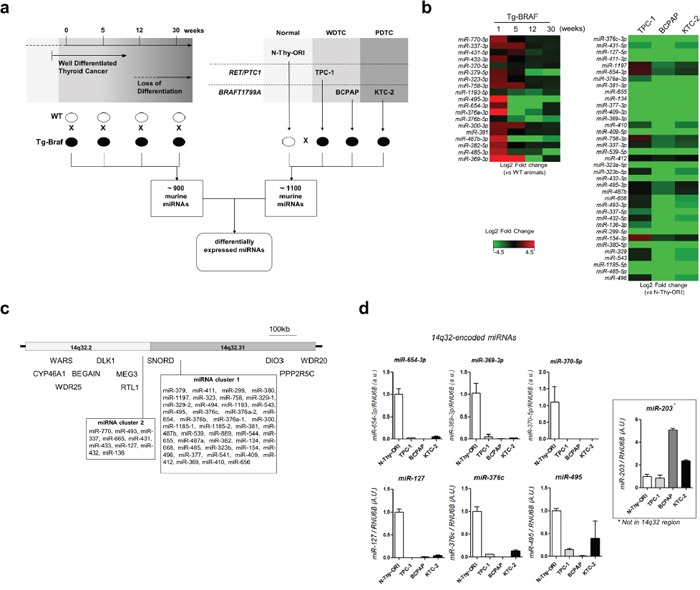
MiRNAs from 14q32 region are down regulated in thyroid cancer cell lines and in murine model of PTC **a**. Large scale analysis of PTC progression *In vivo* and *in vitro*. Panel of thyroid cell lines including normal (N-Thy-ORI), PTC cell line bearing *RET/PTC1* oncogene (TPC-1), and PTC and ATC cell lines bearing *BRAFT1799A* oncogene (BCPAP and KTC-2, respectively). Expression of miRNAs from transgenic animals in each time point was compared with respective wild-type sample. Expression of miRNAs for each thyroid cancer cell line was compared with the normal cell line N-Thy-ORI. **b**. Downregulation of 14q32-encoded miRNAs in Tg-Braf mouse model and thyroid cancer cell lines. Heatmap represents the fold change between each thyroid cancer cell line and normal cells (N-Thy-ORI) and between Tg-Braf and Wild-type animals in each time point. **c**. Schematic representation of DLK1-DIO3 region and miRNA clusters in 14q32 chromosome locus. The distances of 5′ and 3′ flanking genes *CYP46A1, WARS, WRD25, BEGAIN, PPP2R5C* and *WRD20* are not in scale. **d**. Validation of miRNAs from 14q32 region by independent qPCR assay. Increased expression of *miR-203*, which is located downstream of DLK1-DIO3, shows absence of terminal deletion of chromosome 14. *Small RNA U6B* (*RNU6B*) gene was used as endogenous control.

To identify differentially expressed miRNAs during PTC loss of differentiation, we used a transgenic mouse model of PTC [16] that harbors a thyroid-targeted *BRAFT1799A* mutation (Figure 1a). While thyroid glands extracted from 5-weeks old mice exhibit classic well differentiated PTC, thyroid glands from older mice show foci of locally invasive poorly differentiated thyroid carcinoma [17]. Large scale analysis revealed that several miRNAs from the 14q32 genomic region are down-regulated during PTC progression (Figure 1b). Importantly, the miRNAs *miR-495-3p, miR-654-3p, miR-376a-3p* and *miR-487b-3p* exhibited marked downregulation after 5 weeks in contrast to a slight reduction of expression observed for most miRNA genes from this region. Downregulation of miRNAs from the 14q32 locus was also observed in thyroid cancer cell lines, prominently in *BRAFT1799A*-mutated BCPAP and KTC-2 cells, as compared to the normal thyroid cell line N-Thy-ORI (Figure 1b). As shown in Figure 1c, the locus 14q32 comprises 53 miRNA genes divided in two clusters, designated here as “miRNA cluster 1” (the larger cluster) and “miRNA cluster 2” (the smaller cluster). This region also comprises several copies of C/D small nucleolar RNAs (*SNORDs*) *112, 113* and *114*, and the imprinted genes *DLK1, RTL1, MEG3, MEG8* and *DIO3*. The TCGA portal (https://cancergenome.nih.gov) reveals a single patient with homodeletion of the entire 14q32 region without any additional information regarding heterozygosity in thyroid cancer. The analysis of randomly-chosen miRNAs from clusters 1 and 2 (*miR-654-3p, mir-369-3p, miR-495, miR-370-5p, miR-127-5p* and *miR-376c-3p*) in tumor cell lines confirmed their downregulation (Figure 1d). Importantly, the reduced expression of miRNAs from the 14q32 locus could not be due to terminal deletion of the 3′ end of chromosome 14, as the thyroid cancer cell lines expressed increased levels of *miR-203a*, situated ∼3Mb downstream of the 14q32 locus.

**Figure 2:**
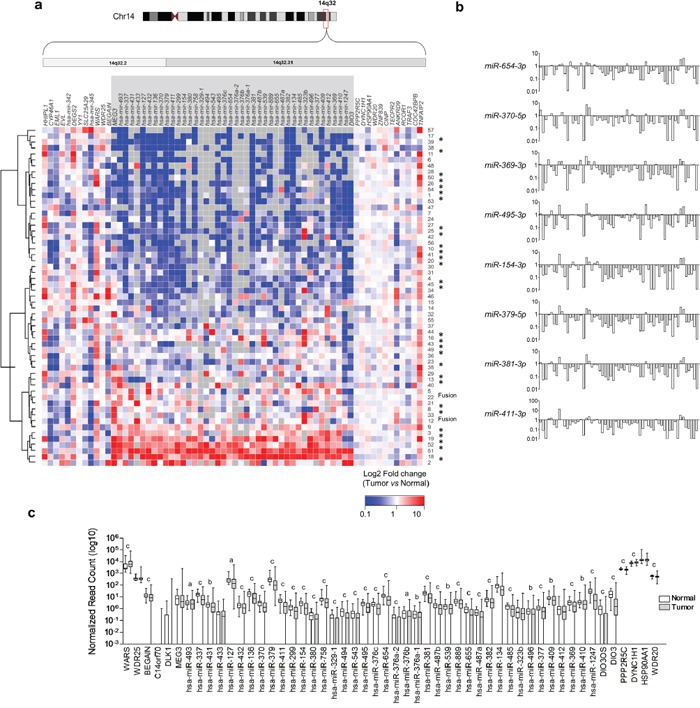
Global downregulation of miRNAs from 14q32-encoded genes in PTC **a**. Gene expression data from 57 paired samples downloaded from TCGA website was used to construct the heatmap. Grey boxes represent null values for sequence read either in Normal or Tumor sample. (*) BRAFT1799A mutation; (Fusion) BRAF gene fusion. **b**. Analysis of 57 paired PTC and normal thyroid tissue reveals concordant miRNA expression pattern across samples. Samples with null values either for PTC or normal tissue were replaced by the lowest value for the given gene. **c**. Average expression of miRNAs from DLK1-DIO3 region and flanking genes in 437 PTC samples compared with 59 normal thyroid samples. (a) p-value <0.05; (b) p-value <0.01; (c) p-value <0.001.

We then analyzed whether downregulation of genes located at the 14q32 genomic region is observed in human PTC samples. We downloaded mRNA and miRNA expression datasets from 57 PTC samples and matched non-tumor thyroid tissue from The Cancer Genome Atlas (TCGA) project, and observed the downregulation of most miRNAs from the 14q32 genomic region (Figure 2a). Importantly, this reduction of expression is restricted to genes situated between *DLK1* and *DIO3*. As shown in Figure 2b, we observed an orchestrated expression pattern for these miRNAs across samples, suggesting either the existence of a polycystronic primary miRNA transcript or regulation of the miRNA cluster by a common mechanism. The analysis of miRNA expression of the whole TCGA dataset, comprising 437 PTC samples and 59 normal thyroid tissue samples, also showed the global miRNAs downregulation located at the 14q32 region (Figure 2c).

### Post-transcriptional regulatory network of miRNAs from the 14q32 region

**Figure 3:**
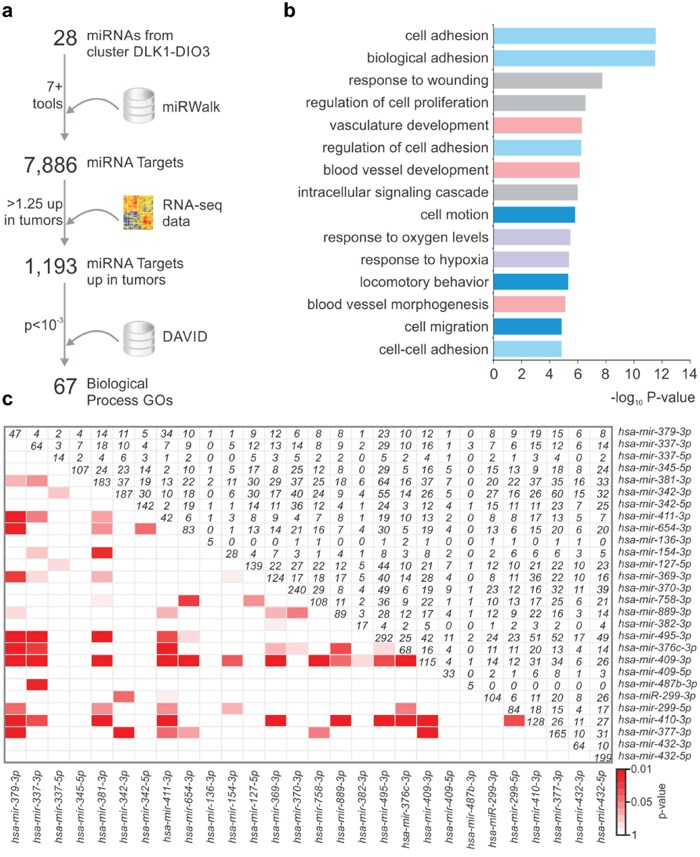
miRNAs from 14q32 region potentially modulate cancer-related processes **a**. The prediction of miRNA targets by at least 7 algorithms was performed using miRWalk webtool. RNA-Seq data was searched for upregulated genes (>1.25 fold) and the resulting list was submited to DAVID webtool. **b**. GO terms enriched among the list of potential targets of 14q32-encoded miRNAs. **c**. miRNAs from DLK1-DIO3 region target common genes. Lower triangular matrix shows the p-value of either Fisher's Exact Test of Chi-square (if sample size is too large for the Fisher test). Upper triangular matrix shows the number of targets shared by both miRNAs. Diagonal numbers are the total number of targets for a given microRNA.

To understand the role of the miRNAs from the *DLK1-DIO3* region in PTC pathogenesis, we analyzed the post-transcriptional regulatory network potentially modulated by these miRNAs *in silico*. As shown in Figure 3a, were selected 28 differentially expressed miRNAs from the 14q32 region that give rise to 32 mature miRNA species. miRNAs with a low or absent number of reads were not considered for analysis. We used the miRWalk webtool to search for predicted targets of each of the 32 mature miRNAs in the 14q32 region. To increase the stringency of target prediction, a minimum of seven algorithms were considered as a cutoff for each miRNA:mRNA interaction. Four mature miRNAs were excluded due to the absence of targets predicted by seven or more algorithms. A total of 7,886 genes targeted by 28 miRNAs were included for Gene Set Enrichment Analysis (GSEA). As the miRNAs from 14q32 are downregulated in PTC, we compared the list of potential targets with RNA-seq V2 data from TCGA and searched for those presenting upregulation (>1.25-fold) in PTC samples. The resulting list of 1,193 predicted targets was subsequently submitted to the DAVID webtool to identify enriched biological processes and signaling pathways. Sixty-seven GO Terms were enriched among the target genes (P value < 0.001; Supplementary Table 2). The most significantly-enriched biological processes were related to cell adhesion and motility, cell proliferation and vascular development (Figure 3b). Additionally, by comparing the lists of miRNA targets, we identified a significant enrichment for 15 out of the 28 miRNAs, suggesting that most of the 14q32-encoded miRNAs may cooperate to regulate the same biological processes (Figure 3c).

### Tumor suppressor properties of miR-654-3p in thyroid cell lines

**Figure 4:**
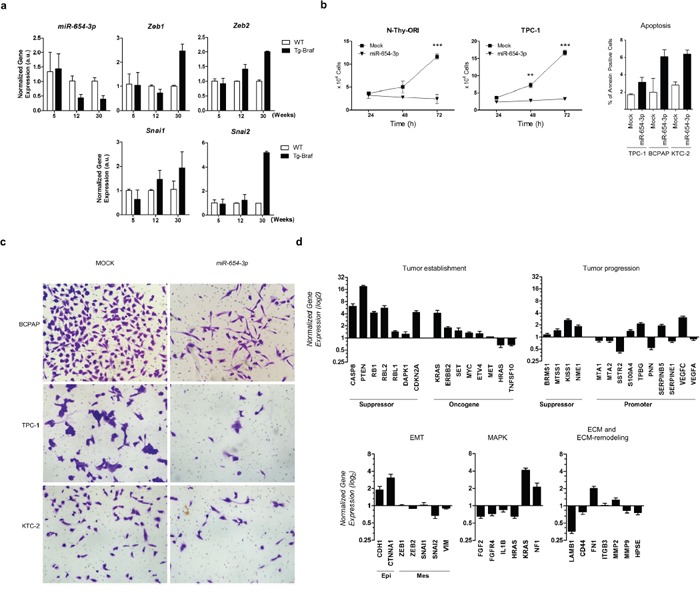
*miR-654-3p* is downregulated during tumor progression of PTC *in vivo* and regulates cell proliferation and migration of PTC *in vitro* **a**. Thyroid tissue extracted from 5-,12- and 30-weeks old animals (Tg-Braf and WT) was homogenized and total RNA extracted was used for cDNA synthesis. *snRNA U6* and *RPL19* were used for normalization of *miR-654-3p and Zeb1 Zeb2, Snai1* and *Snai2* respectively. **b**. N-Thy-ORI and TPC-1 cells were transfected or not with *miR-654-3p* mimetic and after 24, 48, and 72 h, cells were trypsinized and counted. The data is representative of two independent experiments performed in triplicates. **c**. TPC-1, BCPAP and KTC-2 cells transfected or not with *miR-654-3p* mimetic were seeded into the upper compartment of modified Boyden chambers with 8 μm pore inserts as described in Methods section. Representative images are shown. **d**. BCPAP cells were transfected with 30nM *miR-654-3p* mimetic. After 48 h, total RNA was extracted and used for cDNA synthesis. Tumor progression and metastasis genes were profiled using Human Tumor Metastasis qPCR Array (Thermo). *GAPDH, HPRT* and *GUSB* genes were used as endogenous control. Values in Y axis reflect the log_2_ fold change expression levels between BCPAP cells transfected *miR-654-3p* mimetic with those not transfected. (EMT) Epithelial-to-mesenchymal transition; (Epi) Epithelial markers; (Mes) Mesenchymal markers; (ECM) Extra-cellular Matrix. (A-D) (Tg-Braf) Tg-Braf animals; (WT) wild-type animals; (Mock) cells exposed to the transfection reagent only. Bars represent s.d. *P<0.05; **P<0.01; ***P<0,001.

Among the differentially expressed miRNAs from the 14q32 region, we observed that *miR-654-3p* levels decrease with long-term PTC progression in Tg-Braf mice and inversely correlate with the expression of the EMT markers *Zeb1, Zeb2, Snai1 and Snai2* (Figure 4a). The restoration of *miR-654-3p* expression using commercial mimetic miRNA markedly decreased cell proliferation and migration, and increased apoptosis in normal and tumor thyroid cell lines (Figure 4b and 4c). Importantly, a cell migration assay was performed 24 h after *miR-654-3p* transfection, ensuring that the observed effect was due to migration impairment rather than decreased proliferation.

To evaluate the participation of *miR-654-3p* in PTC progression, we analyzed the impact of ectopic expression of *miR-654-3p* on the expression of several genes involved in tumor establishment and progression using a Taqman® Human Tumor Metastasis qPCR Array. The *miR-654-3p*-transfected cells showed increased expression levels of the epithelial cell markers *CDH1* and *CTNNA1* and decreased levels of *SNAI2*, with no significant changes in *ZEB1, ZEB2, SNAI1* and *VIM* levels (Figure 4d). No morphological changes were observed through light microscopy after transfection of *miR-654-3p* (data not shown). Known tumor suppressor genes were upregulated after transfection of mimetic *miR-654-3p*, including *CASP8, PTEN, DAPK1, RB1* and the cell cycle regulator *CDKN2A*. Moreover, key genes involved in cell adhesion, migration and ECM remodeling were downregulated after transfection of mimetic *miR-654-3p*, such as *LAMB1* and *MMP9*. Also, decreased levels of *HRAS, FGF2, FGFR4* and *IL1B*, as well as increased *NF1*, components of MAPK signal transduction, were observed after transfection of *miR-654-3p*. Importantly, restoration of *miR-654-3p* upregulated the expression of the metastasis-suppressor genes *BRMS1, MTSS1, KISS1* and *NME1*, and downregulated the expression of several genes related to tumor progression and metastasis, including *MTA1, MTA2, SERPINE1, SSTR2* and *CD44*. Altogether, these data suggest that restoration of *miR-654-3p* could contribute to a more differentiated, less aggressive phenotype in PTC.

## DISCUSSION

Although the abnormal expression of miRNAs has been widely described in well-differentiated thyroid cancer, the role of these molecules in PTC progression remains unclear. *In silico* and experimental analyses suggest that abnormal miRNA expression may participate in key biological processes during cancer invasion and dissemination, and can contribute to aggressive behavior of PTC [10, 18–20]. Using an *in vivo* mouse model of PTC, we identified marked downregulation of miRNAs from the 14q32 region concomitant to loss of differentiation and EMT. PTCs generated in Tg-Braf animals recapitulate the intra-tumor heterogeneity observed in a fraction of PTC patients, including loss of differentiation, TGFβ-driven EMT, tumor-associated macrophages and local invasiveness [17]. Riesco-Eizaguirre and colleagues have shown that *BRAFT1799A* mutation induces repression of thyroid-differentiation gene *NIS* through the activation of a TGF-β autocrine loop, leading to radioiodine resistance and poor prognosis [21]. We have shown that miRNAs from 14q32 region target EMT-related genes, which could contribute with the aggressive phenotype observed in these tumors.

Although the deregulation of select individual miRNAs from the 14q32 region has been shown in PTC cell lines [22, 23], large scale analysis of miRNA expression *in vivo* combined with data mining of the TCGA project allowed us to identify the synchronized downregulation of miRNAs limited to the 14q32 region spanning from the *DLK1* to *DIO3* genes, also known as the *DLK1-DIO3* region. The coordinated downregulation of miRNAs from the 14q32 region in PTC is aligned with the observation of a large polycistronic transcript comprising the largest miRNA cluster in the human genome [24, 25].

Several studies have shown downregulation of miRNAs from the 14q32 region in different types of cancer, such as ovarian, breast and prostate cancer, and gastrointestinal stromal tumor, [26–29], with significant correlations to poor prognosis and aggressiveness. Importantly, a tumor suppressor role has been recognized in several of the downregulated miRNAs from the 14q32 region through the targeting of key oncogenes in glioblastoma, neuroblastoma, metastatic lung cancer, hepatic cancer, pituitary adenoma and rhabdomyosarcoma [30–34]. For example, restoration of *miR-370* expression led to downregulation of *FOXM1* in acute myeloid leukemia, promoting cell growth arrest and senescence [35]. In contrast, miRNAs from the 14q32 region may act as oncogenes as well [36–38], suggesting that these miRNAs may have different biological roles depending on the tissue of origin and genetic background.

The mechanisms underlying modulation of this region remain to be fully understood. In this study, increased expression of *miR-203* observed in thyroid cancer cell lines excludes the existence of terminal deletion of chromosome 14. TCGA data shows homodeletion of the 14q32 region in one patient sample, however without heterozygosis status. Additionally, despite a large number of oncogenic mutations that have been recently identified in poorly differentiated (PDTC) and anaplastic thyroid carcinomas (ATC), no genetic alteration spanning the 14q32 region was observed [39]. The relationship between MAPK pathway and the 14q32 region remains uncertain. Cahill and colleagues have shown that human derived *BRAFT1799A-* and *RET/PTC*-bearing thyroid tumor cells, KAT10 and TPC-1 respectively, express lower levels of 14q32-encoded miRNAs *miR-323-3p, miR-370-5p, miR-127-3p, miR-299* and *miR-154* [22, 23]. However, the *in vitro* acute transfection of each oncogene into the normal thyroid cell line N-Thy-ORI did not show the same level of impairment of expression of these miRNAs. This observation, combined with the long-term (12 weeks) downregulation of 14q32-encoded miRNAs during progression of PTC in Tg-Braf mice suggests that other mechanisms than the driver oncogene activation may play an additional role on the silencing of 14q32 genes. Recent studies have shown that epigenetic changes, such as DNA methylation and chromatin remodeling by lncRNA-mediated mechanisms, may participate in regulating the expression of the 14q32-encoded miRNAs [28, 40]. Also, imprinting imbalance could lead to the differential modulation of paternally and maternally expressed genes from the 14q32 region and could explain, at least in part, the increased levels of *DIO3* observed in some PTC samples [41, 42]. Altogether, these data suggest that multiple mechanisms other than genetic mutations or chromosomal loss might be involved in the regulation of 14q32-encoded miRNAs in thyroid cancer.

Due to the large number of miRNAs present in the 14q32 region that are concomitantly deregulated, the miRNA:target interaction pattern may lead to a complex regulatory network that may include thousands of potential targets. Our GSEA (Figure 3) indicates that miRNAs from the 14q32 region could contribute to tumor progression and metastasis by targeting key regulators of biological processes involved in tumorigenesis and tumor progression, such as cell adhesion and migration, proliferation, hypoxic response and wound healing. *miR-654-3p*, which has been described as a tumor suppressor miRNA in prostate cancer cell lines [28], is downregulated during PTC progression in the Tg-Braf model and in human PTC samples. Our functional analysis indicates that *miR-654-3p* markedly decreases cell proliferation and increases apoptosis, possibly by restoring the expression of key tumor suppressor genes. Although no morphological changes were observed, transfection of *miR-654-3p* increased the expression of *CDH1* and *CTNNA1*, and decreased the expression of *SNAI2*, which is consistent with an “epithelial” genetic program. Importantly, the levels of key modulators of tumor progression, such as ECM and ECM-remodeling genes, as well as metastasis-suppressing and -promoting genes, were restored after transfection of a *miR-654-3p* mimetic.

In conclusion, we have identified the global downregulation of miRNAs from 14q32 region in a mouse model of PTC progression and in human PTC samples. The potential network modulated by these miRNAs may contribute to important aspects of cancer development and progression, such as cell adhesion, migration, angiogenesis and proliferation. Finally, *in vitro* functional analyses highlight tumor suppressor properties for *miR-654-3p* in PTC. Due to the large number of potential key cancer-related targets, further functional analyses of miRNAs from the 14q32 region may be of great interest for the development of new therapeutic strategies for thyroid cancer.

## MATERIALS AND METHODS

### Cell lines and animals

The PTC-derived cell line TPC-1, which spontaneously harbors RET/PTC1 rearrangement, was kindly provided by Dr James A Fagin (Human Oncology and Pathogenesis Program, Memorial Sloan-Kettering Cancer Center, New York, NY, USA). BCPAP, derived from *BRAFT1799A*-positive PTC was kindly donated by Dr Massimo Santoro (Medical School, University ‘Federico II’ of Naples, Naples, Italy). The ATC-derived cell line KTC-2, also harboring *BRAFT1799A* oncogene was kindly provided by Dr. Norisato Mitsutake (Nagasaki University Graduate School of Biomedical Sciences, Nagasaki, Japan). The origin and genetic background of these cell lines has been recently authenticated [43, 44]. Immortalized non-tumoral thyroid follicular cells N-Thy-ORI were purchased from Sigma and maintained in RPMI (Invitrogen) supplemented with 10% FBS and 2 mM L-glutamine (Invitrogen). TPC-1 and BCPAP cells were cultivated as previously described [45]. KTC-2 cells were maintained in RPMI supplemented with 5% FBS. All cell lines were cultivated with 100 U/ml penicillin, 1μg/ml streptomycin and 100μg/ml amphotericin at 37°C and in a 5% CO2 atmosphere.

FVB transgenic mice, harboring the thyroid-targeted expression of *BRAFT1799A* oncogene [16] as well as their wild-type counterparts were donated by Dr James Fagin (Human Oncology and Pathogenesis Program, Memorial Sloan-Kettering Cancer Center, New York, NY, USA), and FVB WT mice were used as control. Animal experimentation was conducted in accordance with guidelines from the Ethical Committee of the Institute of Biomedical Sciences (n#134/ p93/ b2), University of São Paulo, Brazil.

### Gene expression analysis

For the large scale analysis of miRNA expression, thyroid glands were surgically removed from euthanized animals at different time points (1, 5, 12 and 30 weeks) and embedded in RNA Later (Ambion). Total RNA was phenol-chloroform-extracted using TRIzol reagent (Invitrogen) from homogenized thyroid tissue samples according to the manufacturer's instructions. Small RNA fraction was purified using miRNeasy kit (Qiagen) and integrity was verified using Agilent small RNA chip in Agilent Bioanalyzer (Agilent). MiRNA expression quantification was performed using miScript II RT kit for reverse transcription and murine miScript PCR Arrays (Qiagen) for mature miRNA amplification, according to manufacturer's instructions. Data analysis was performed using the online software *PCR Array Data Analysis Web Portal* (http://pcrdataanalysis.sabiosciences.com/mirna/arrayanalysis.php). The melting curve of each amplification product was manually checked to assure the specificity of the reaction. In order to increase reliability, the standard deviation for the Ct values of the 5 genes provided as endogenous controls were calculated and the endogenous gene with the highest value was discarded.

For quantification of miRNA and mRNA expression *in vitro*, 1×10^5^ cells were seeded into 60 mm dishes. Forty-eight hours after transfection of miRNA mimetic, total RNA was phenol-chloroform-extracted using TRIzol reagent (Invitrogen) according to the manufacturer's instructions. For miRNA expression analysis 10 ng of total RNA was reverse transcribed using TaqMan® MicroRNA Reverse Transcription kit (Applied Biosystems) according to the manufacturer's instructions. MiRNA expression was detected using TaqMan Universal PCR Master Mix No AmpErase UNG (Applied Biosystems). For the quantification of mRNA expression 2 μg of total RNA was reverse transcribed using MMLV Reverse transcription kit (Life). *RNA U6 small nuclear 2* (*RNU6B*) and *RPL19* genes were used as endogenous controls for normalization of miRNA and mRNA input, respectively. The expression of metastasis-related genes was quantified using Human Tumor Metastasis Taqman® Array plate (Thermo-Fischer), according with manufacturer's instructions. All amplification reactions were performed using universal cycling conditions in ViiA-7 Real-Time PCR System (Applied Biosystems). RT primers part numbers for miRNA (Applied Biosystems) and the oligonucleotides used for qPCR reaction are shown in Supplementary Table 1. The differential gene expression was calculated according with Pfafll [46].

### Data mining of the Cancer Genome Atlas

miRNA (miRNASeq) and mRNA (RNASeqV2) Gene expression, Copy Number Variation (CNV Lowpass DNA-Seq) and clinical data were downloaded in “level 3” format from TCGA web portal (https://cancergenome.nih.gov). miRNA and mRNA differential expression was calculated using Normalized Read count data for each individual sample. For miRNA and mRNA expression analysis, transcripts were considered not valid when 42 or more (75%) pairs, out of 57 PTC *versus* matched normal tissue, had not valid fold changes. In total, 579 out of 1,046 miRNAs were excluded and 467 were considered valid. Also, mRNA transcripts whose median expression were <= 5 reads across all 114 samples (57 PTC and paired normal tissue) were excluded (5,142 out of 20,502).

### Gene Set Enrichment Analysis

The miRNA-target prediction was performed using miRWalk program (http://www.umm.uni-heidelberg.de/apps/zmf/mirwalk/), accessing a total of 12 algorithms. To increase stringency, one interaction was considered valid when predicted by TargetScan algorithm and 7 or more algorithms. The list of potential targets was then compared with RNA-seq dataset downloaded from TCGA. Upregulated (> 1.25 fold) mRNA targets were selected and submitted to DAVID web tool (https://david.ncifcrf.gov/). Biological processes enriched among the target genes potentially targeted by miRNAs from region 14q32 were identified. The enrichment of common targets between each miRNA from 14q32 region was calculated using Fisher's Exact Test or Fisher's Exact Test of Chi-square, if sample size is too large for the Fisher test.

### Transient transfection of miR-654-3p mimetic

Normal and tumor thyroid cells were transfected with *miR-654-3p* miRVana mimic (Life Technologies) at concentration of 10-30nM. The concentration of 30nM was proved to be more efficient and used for further experiments. Transfection was performed 24h after seeding using Lipofectamine 2000® (Invitrogen). Cells exposed to transfection reagent only were used as control and denominated Mock.

### Cell proliferation, migration and apoptosis

Cell counting, migration and apoptosis analyses were performed as previously described, with modifications [45, 47]. Growth curve and apoptosis were measured using Guava EasyCyte Mini Flow Cytometer (Guava Technologies). Migrating cells were visualized after staining with 0.5% Crystal Violet in Ethanol in Nikon Eclipse E600 microscope.

### Statistical analysis

Statistical analysis was performed using GraphPad Prism Software version 5.00 (San Diego, CA, USA). After the test for normality using Kolmogrov-Smirnov test, samples were compared using non-parametric Mann-Whitney test, when they presented non-Gaussian distribution, and using Students t test, when they p-resented Gaussian distribution. Differences were considered significant when P<0.05.

## SUPPLEMENTARY TABLES




