# Supplementary material for: Down-regulation of 14q32-encoded miRNAs and tumor suppressor role for miR-654-3p in papillary thyroid cancer
Source: Oncotarget. 2016 Dec 24;8(6):9597–607. doi: 10.18632/oncotarget.14162 (PMC5354756; doi:10.18632/oncotarget.14162)
Supplement: Supplementary file 1 [file oncotarget-08-9597-s001.pdf]

Down-regulation of 14q32-encoded miRNAs and tumor suppressor role for *miR-654-3p* in papillary thyroid cancer

SUPPLEMENTARY TABLES

Supplementary Table 1: Gene set enrichment analysis of predicted targets of 14q32-encoded miRNAs. The 67 significantly enriched processes are displayed.

See Supplementary File 1

Supplementary Table 2: mRNA and miRNA Oligonucleotide information

Gene ID	Sequence (5' - 3')
ZEB1_hum_F	GATGACCTGCCAACAGACCA
ZEB1_hum_R	GCCCTTCCTTTCCTGTGTCA
ZEB2_hum_F	AGTGTGCCCAACCATGAGTC
ZEB2_hum_R	TCCTTCATTTCTTCTGGACCATC
Zeb2_mous_F	GAAAAGCAGTTCCCTTCTGC
Zeb2_mous_R	GCTCGATAAGGTGGTGTTTG
Zeb1_mous_F	AAGAGCAGCTCACTGTTGAGAC
Zeb1_mous_R	GATAGGGCTTTTCCCCAGAG
VIM_hum_F	GGCTCGTCACCTTCGTGAAT
VIM_hum_R	GAGAAATCCTGCTCTCCTCGC
SNAI1_hum_F	TCTAGGCCCTGGCTGCTAC
SNAI1_hum_R	TCTTGACATCTGAGTGGGTCTG
SNAI2_hum_F	TCTGCAGACCCATTCTGATG
SNAI2_hum_R	TCCTCATGTTTGTGCAGGAG
PTEN_hum_F	GCCAACCGATACTTTTCTCC
PTEN_hum_R	GCTAGCCTCTGGATTTGACG
NF1_hum_F	GAGGCCAGTGTTGTGTTTCC
NF1_hum_R	CAAATTTGGATCTTGGCACA
RPL19_hum_F	TCTCATGGAACACATCCACAA
RPL19_hum_R	TGGTCAGCCAGGAGCTTCTT
Rpl19_mous_F	CCTGAAGGTCAAAGGGAATG
Rpl19_mous_R	TCGTGCTTCCTTGGTCTTAG

miRNA ID	Life Technologies Part Number
hsa-miR-654-3p	PN002239
hsa-miR-370-5p	PN000558
hsa-miR-369-3p	PN000557
hsa-miR-495-3p	PN001108
hsa-miR-376c	PN002122
hsa-miR-127-5p	PN002229
hsa-miR-203-3p	PN000507
RNU6B	PN4427975
