# Supplementary material for: Down-regulation of 14q32-encoded miRNAs and tumor suppressor role for miR-654-3p in papillary thyroid cancer
Source: Oncotarget. 2016 Dec 24;8(6):9597–607. doi: 10.18632/oncotarget.14162 (PMC5354756; doi:10.18632/oncotarget.14162)
Supplement: Supplementary file 2 [file oncotarget-08-9597-s002.docx]

**Supplementary table 1. Gene set enrichment analysis of predicted targets of 14q32-encoded miRNAs.** The 67 significantly enriched processes are displayed.

| *Term* | | *Count* | *%* | *PValue* | *FDR* | *Fold Enrich.* |
| --- | --- | --- | --- | --- | --- | --- |
| GO:0007155 | cell adhesion | *100* | 8.39 | *2.78E-12* | 5E-09 | 2.07 |
| GO:0022610 | biological adhesion | *100* | 8.39 | *3.03E-12* | 6E-09 | 2.07 |
| GO:0009611 | response to wounding | *73* | 6.12 | *1.79E-08* | 3E-05 | 2.00 |
| GO:0042127 | regulation of cell proliferation | *93* | 7.80 | *2.84E-07* | 5E-04 | 1.71 |
| GO:0001944 | vasculature development | *41* | 3.44 | *5.11E-07* | 9E-04 | 2.37 |
| GO:0030155 | regulation of cell adhesion | *28* | 2.35 | *5.67E-07* | 1E-03 | 2.96 |
| GO:0001568 | blood vessel development | *40* | 3.36 | *7.29E-07* | 1E-03 | 2.37 |
| GO:0007242 | intracellular signaling cascade | *131* | 10.99 | *9.95E-07* | 2E-03 | 1.51 |
| GO:0006928 | cell motion | *62* | 5.20 | *1.58E-06* | 3E-03 | 1.89 |
| GO:0070482 | response to oxygen levels | *27* | 2.27 | *3.39E-06* | 6E-03 | 2.78 |
| GO:0001666 | response to hypoxia | *26* | 2.18 | *4.18E-06* | 8E-03 | 2.81 |
| GO:0007626 | locomotory behavior | *41* | 3.44 | *4.89E-06* | 9E-03 | 2.17 |
| GO:0048514 | blood vessel morphogenesis | *34* | 2.85 | *7.57E-06* | 1E-02 | 2.34 |
| GO:0016477 | cell migration | *40* | 3.36 | *1.40E-05* | 3E-02 | 2.10 |
| GO:0016337 | cell-cell adhesion | *40* | 3.36 | *1.40E-05* | 3E-02 | 2.10 |
| GO:0007267 | cell-cell signaling | *68* | 5.70 | *5.43E-05* | 1E-01 | 1.64 |
| GO:0007610 | behavior | *56* | 4.70 | *6.95E-05* | 1E-01 | 1.73 |
| GO:0051674 | localization of cell | *41* | 3.44 | *7.16E-05* | 1E-01 | 1.94 |
| GO:0048870 | cell motility | *41* | 3.44 | *7.16E-05* | 1E-01 | 1.94 |
| GO:0048666 | neuron development | *44* | 3.69 | *7.31E-05* | 1E-01 | 1.88 |
| GO:0051094 | positive regulation of developmental process | *38* | 3.19 | *8.47E-05* | 2E-01 | 1.98 |
| GO:0007167 | enzyme linked receptor protein signaling pathway | *44* | 3.69 | *9.02E-05* | 2E-01 | 1.87 |
| GO:0006935 | chemotaxis | *26* | 2.18 | *9.51E-05* | 2E-01 | 2.36 |
| GO:0042330 | taxis | *26* | 2.18 | *9.51E-05* | 2E-01 | 2.36 |
| GO:0043588 | skin development | 10 | 0.84 | *9.58E-05* | 2E-01 | 5.00 |
| GO:0043068 | positive regulation of programmed cell death | *52* | 4.36 | *1.14E-04* | 2E-01 | 1.74 |
| GO:0006954 | inflammatory response | *42* | 3.52 | *1.20E-04* | 2E-01 | 1.87 |
| GO:0010942 | positive regulation of cell death | *52* | 4.36 | *1.27E-04* | 2E-01 | 1.73 |
| GO:0006979 | response to oxidative stress | *26* | 2.18 | *1.42E-04* | 3E-01 | 2.30 |
| GO:0000302 | response to reactive oxygen species | *16* | 1.34 | *1.53E-04* | 3E-01 | 3.09 |
| GO:0007169 | transmembrane receptor protein tyrosine kinase signaling pathway | *32* | 2.68 | *1.53E-04* | 3E-01 | 2.07 |
| GO:0043536 | positive regulation of blood vessel endothelial cell migration | 6 | 0.50 | *1.53E-04* | 3E-01 | 9.67 |
| GO:0007568 | aging | *20* | 1.68 | *1.70E-04* | 3E-01 | 2.64 |
| GO:0043065 | positive regulation of apoptosis | *51* | 4.28 | *1.80E-04* | 3E-01 | 1.72 |
| GO:0010648 | negative regulation of cell communication | *34* | 2.85 | *2.02E-04* | 4E-01 | 1.99 |
| GO:0009968 | negative regulation of signal transduction | *31* | 2.60 | *2.73E-04* | 5E-01 | 2.03 |
| GO:0042060 | wound healing | *28* | 2.35 | *2.82E-04* | 5E-01 | 2.13 |
| GO:0009967 | positive regulation of signal transduction | *38* | 3.19 | *2.85E-04* | 5E-01 | 1.87 |
| GO:0007569 | cell aging | 10 | 0.84 | *2.87E-04* | 5E-01 | 4.39 |
| GO:0007346 | regulation of mitotic cell cycle | *24* | 2.01 | *2.89E-04* | 5E-01 | 2.29 |
| GO:0010647 | positive regulation of cell communication | *41* | 3.44 | *3.17E-04* | 6E-01 | 1.81 |
| GO:0042542 | response to hydrogen peroxide | *13* | 1.09 | *3.52E-04* | 6E-01 | 3.37 |
| GO:0051726 | regulation of cell cycle | *41* | 3.44 | *3.59E-04* | 7E-01 | 1.80 |
| GO:0031175 | neuron projection development | *34* | 2.85 | *3.64E-04* | 7E-01 | 1.93 |
| GO:0010035 | response to inorganic substance | *29* | 2.43 | *3.89E-04* | 7E-01 | 2.05 |
| GO:0030030 | cell projection organization | *44* | 3.69 | *4.53E-04* | 8E-01 | 1.73 |
| GO:0043535 | regulation of blood vessel endothelial cell migration | 7 | 0.59 | *4.62E-04* | 8E-01 | 6.34 |
| GO:0030182 | neuron differentiation | *50* | 4.19 | *5.14E-04* | 9E-01 | 1.66 |
| GO:0014070 | response to organic cyclic substance | *20* | 1.68 | *5.94E-04* | 1E+00 | 2.40 |
| GO:0045321 | leukocyte activation | *32* | 2.68 | *6.03E-04* | 1E+00 | 1.92 |
| GO:0022604 | regulation of cell morphogenesis | *21* | 1.76 | *6.25E-04* | 1E+00 | 2.32 |
| GO:0007156 | homophilic cell adhesion | *21* | 1.76 | *6.25E-04* | 1E+00 | 2.32 |
| GO:0051130 | positive regulation of cellular component organization | *26* | 2.18 | *6.62E-04* | 1E+00 | 2.08 |
| GO:0043067 | regulation of programmed cell death | *81* | 6.80 | *6.67E-04* | 1E+00 | 1.45 |
| GO:0045785 | positive regulation of cell adhesion | *13* | 1.09 | *6.85E-04* | 1E+00 | 3.14 |
| GO:0001775 | cell activation | *36* | 3.02 | *6.93E-04* | 1E+00 | 1.82 |
| GO:0000904 | cell morphogenesis involved in differentiation | *32* | 2.68 | *6.94E-04* | 1E+00 | 1.90 |
| GO:0010941 | regulation of cell death | *81* | 6.80 | *7.47E-04* | 1E+00 | 1.44 |
| GO:0051270 | regulation of cell motion | *27* | 2.27 | *7.64E-04* | 1E+00 | 2.03 |
| GO:0009891 | positive regulation of biosynthetic process | *71* | 5.96 | *7.93E-04* | 1E+00 | 1.48 |
| GO:0045859 | regulation of protein kinase activity | *41* | 3.44 | *8.21E-04* | 1E+00 | 1.72 |
| GO:0001558 | regulation of cell growth | *27* | 2.27 | *8.26E-04* | 2E+00 | 2.02 |
| GO:0008284 | positive regulation of cell proliferation | *47* | 3.94 | *8.73E-04* | 2E+00 | 1.65 |
| GO:0034097 | response to cytokine stimulus | *15* | 1.26 | *9.09E-04* | 2E+00 | 2.75 |
| GO:0007243 | protein kinase cascade | *43* | 3.61 | *9.41E-04* | 2E+00 | 1.69 |
| GO:0007411 | axon guidance | *18* | 1.51 | *9.88E-04* | 2E+00 | 2.44 |
